# Antifungal and antioxidant activities of *Coleonema album* and *C. pulchellum* against skin diseases

**DOI:** 10.1080/13880209.2017.1296470

**Published:** 2017-03-05

**Authors:** Olufunke O. Fajinmi, Jiří Grúz, Petr Tarkowski, Manoj G. Kulkarni, Jeffrey F. Finnie, Johannes Van Staden

**Affiliations:** a Research Centre for Plant Growth and Development, School of Life Sciences, University of KwaZulu-Natal Pietermaritzburg, Scottsville, South Africa;; b Laboratory of Growth Regulators and Department of Chemical Biology and Genetics, Centre of the Region Haná for Biotechnological and Agricultural Research, Faculty of Science, Palacký University and Institute of Experimental Botany, Olomouc, Czech Republic;; c Centre of the Region Haná for Biotechnological and Agricultural Research, Central Laboratories and Research Support, Faculty of Science, Palacký University, Olomouc, Czech Republic;; d Centre of the Region Haná for Biotechnological and Agricultural Research, Department of Genetic Resources for Vegetables, Medicinal and Special Plants, Crop Research Institute, Olomouc, Czech Republic

**Keywords:** Atopic dermatitis, *Trichophyton rubrum*, *Trichophyton mentagrophytes*

## Abstract

**Context:***Coleonema album* (Thunb) Bart. & H. L. Wendl (Rutaceae) has been used in the formulation of skincare products, and the *Khoisan* people rub it on their skin to add luster. *Coleonema pulchellum* I. Williams has received less attention in the South African traditional medicine.

**Objective:** This study investigates the antifungal and antioxidant activities of *C. album* and *C. pulchellum* essential oil (EO) and leaf extracts; and analyzes the chemical components of their EOs.

**Materials and methods:** Antifungal activity of leaf extracts was determined using the microdilution method with griseofulvin and ketoconazole as controls. Antifungal capacity of EO was investigated using the ‘Volatile release plate method’. *Trichophyton rubrum* (ATCC 28188) and *T. mentagrophytes* (ATCC 9533) mycelia (0.3 cm diameter) were placed on fresh yeast malt agar in Petri dishes with filter paper (impregnated with 20 μL of EO) on the lid for direct exposure to EO volatiles while plates without EO were used as controls. The incubation time was seven days. Antioxidant activities of the leaf extracts were determined.

**Results:** Methanol leaf extract of *C. pulchellum* inhibited the growth of three fungi tested with MIC values of 195, 391 and 49 μg/mL for *Trichophyton rubrum*, *Trichophyton mentagrophytes* and *Microsporum gypseum,* respectively. Terpenes formed the major components of the EO. The EO from both plants inhibited the growth of *T. rubrum in vitro.*

**Discussion and conclusion:** This study revealed the therapeutic value of *C. pulchellum*. *Coleonema album* and *C. pulchellum* should be considered as potential plants for skin ointment from natural origin.

## Introduction

Fungal infections of the skin and nails represent a major group of mycoses (Havlickova et al. 2008) and are among the most common skin disease manifestation in HIV/AIDS patients. They can occur at any stage of the disease and some of the dermatoses are unique to HIV infection while others are common conditions, which also occur in HIV-negative individuals (National Aids and STI Control Program 2008). Patients with HIV infection exhibit a wide range of skin pathology which includes bacterial, fungal and viral infections; skin tumours; inflammatory and eczematous eruptions; and drug rashes (Rudikoff 2002). In a study involving 186 HIV positive patients, 175 (94%) suffered from one or more cutaneous disorders (Ho & Wong 2001). Dermatophytoses manifests as an opportunistic infection which is four times more prevalent in Acquired Immune Deficiency Syndrome (AIDS) patients (Goodman et al. 1987).

The frequently isolated species are *Trichophyton rubrum, T. mentagrophytes* and *Epidermophyton floccosum* (Fernandes et al. 1998). *Microsporum gypseum* is a geophillic dermatophyte, which is rarely isolated from skin lesions of immunocompromised patients (Bhagra et al. 2013). *Microsporum gypseum* may cause *Tinea capitis* (ringworm of the scalp and hair) and *Tinea corporis* (ringworm of the glabrous skin) in immunocompetent hosts (Pandey & Pandey 2013). The most common skin disorder identified in HIV/AIDS individuals is fungal infection (Tse 2007).

The increased incidence of resistance of skin fungal diseases to antibiotics is of great concern. The situation is more prominent among HIV/AIDS individuals, thus adding to the burden of skin diseases in Africa as a region. Owing to the high incidence of HIV/AIDS in South Africa, there is an urgent need for the development of new medicines to cure various HIV/AIDS opportunistic infections, especially skin diseases. The improvement of previously used antibiotics, and the introduction of a new range of antibiotics for the treatment of skin diseases of fungal origin, has not been able to significantly solve the problem of the increasing incidence of skin diseases resistant to antibiotics. Hence, the search for an effective antifungal skin ointment of natural origin could present a lasting solution to the shortcomings of the various antibiotics used in clinical practice. Rutaceae plants are known to be a source of coumarins, terpenes, flavonoids and other therapeutic agents that have been reported to be effective in the treatment of skin diseases and alleviation of oxidative stress and inflammation. Scientific literature has shown a link between inflammation and oxidative stress as it plays a crucial role in the pathogenesis of chronic inflammatory diseases such as atopic dermatitis (AD) (eczema/ringworm).

Restoration of the redox balance through the use of antioxidants from natural origin could be a ground-breaking approach in the treatment of inflammatory skin conditions. Natural products offer a variety of anti-inflammatory (Yasser & Nabil 2012) and antioxidant agents (Kostova 2006), which can counter the oxidative stress, dryness, scaling, and inflammations that comes into play during the pathogenesis of skin diseases.

A combination of inflammatory conditions and an overwhelmed antioxidant system could result in pathological levels of Reactive Oxidative Species (ROS) and thus, oxidative stress aggravates the disease state (Wagener et al. 2013). Kruk and Duchnik (2014) reported how oxidative stress plays a crucial role in the pathogenesis of dermatologic diseases through the disruption of the defensive systems against ROS/RNS (Reactive Nitrogen Species). ROS are produced during normal metabolic processes and are an integral part of the normal cellular function, usually of little harm as the body’s defence system reduces their damaging effects (Trouba et al. 2002). A higher percentage of oxidative stress in AD is caused as a result of the increase in lipid peroxidation and decreased levels of antioxidants (Sivaranjani et al. 2013). Antioxidants shield the body from the damaging effects of ROS and can inhibit and/or reverse several processes that contribute to epidermal toxicity and skin diseases (Trouba et al. 2002).

*Coleonema* (Rutaceae) is a genus of flowering plants, and all eight known species are endemic to the Cape region of South Africa, but they have been domesticated in some other countries such as Australia. All the species of the genus are characterized with dotted oil glands on their leaves. The main chemical components of *Coleonema album* (Thunb) Bart. & H. L. Wendl oil are myrcene, phellandrene, pinene, ocimene, and germacrene-D (Başer et al. 2006). A wide range of coumarin derivatives have been isolated from *C. album* (Dreyer et al. 1972; Gray 1981). Pharmacological activities of *C. album* have been attributed to its terpenes and coumarins (and coumarin derivatives). These include: antithrombotic and antiplatelet (Hoult & Paya 1996), antimicrobial (Kayser & Kolodziej 1999; Laurin et al. 1999; Lis-Balchin & Hart 2002; Okunade et al. 2004; Esterhuizen et al. 2006a; Liebenberg 2008) and antioxidant (Esterhuizen et al. 2006b; Liebenberg 2008) activities.

A tincture made from *C. album* and marketed as ‘*Immunat*’ is widely used in South Africa as an herbal remedy to build the body’s immune system. Some compounds isolated from this plant have activity against several inflammatory mediators (Eldeen & Van Staden 2008). Even though ethnobotanical work has been done on *C. album* (Fajinmi et al. 2013), the pharmacological activities of its sister species (*C. pulchellum* I. Williams) is yet to be fully explored. Fishermen traditionally rub their hands on the leaves to remove the fishy smell of bait (Van Wyk & Gericke 2000). Essential oil (EO) derived from *C. album* is incorporated into several skin care products. Fajinmi et al. (2014) reported that there is currently an increase in demand for and utilization of *C*. *album* in South African traditional medicine. Many South African natural product companies are now pursuing export markets for *C. album* oil, which may put pressure on the plant’s natural populations in the near future (Fajinmi et al. 2013).

However, its sister species, *C. pulchellum* has received little or no attention. Apart from the development of an effective propagation technique and control of harvesting of *C. album* wild populations, the use of another member of the genus with the same medicinal value can help reduce the pressure on *C. album* plants and thus prevent the plant from becoming an endangered species in the near future. *Coleonema album* and *C. pulchellum* have similar morphological features in the field and can only be distinguished by the colour of their flowers which are white and pink, respectively. However, some hybrids (with pink flowers) of both plants exist in gardens and nurseries in South Africa. The aim of this study was to investigate the *in vitro* activity of *C. album* and *C. pulchellum* leaf extract, EO volatiles against three fungal strains responsible for AD, analyze the bioactive compounds present in the EO of both species and investigate the antioxidant activity of the leaf extracts.

## Materials and methods

### Antifungal assay of leaf extracts

Leaves were sourced from the KwaZulu-Natal botanical garden (Pietermaritzburg, South Africa) in September 2014. The plant was verified by the botanical garden horticulturist (Ms Alison Young). Voucher specimen, *Coleonema album* O Fajinmi 01 and *Coleonema pulchellum* O Fajinmi 21 have been deposited in the University of KwaZulu-Natal Herbarium. Fresh leaves were dried in an incubator at 40 °C for four days. Dried leaves were ground into powder and stored in air-tight containers at 23 ± 1 °C in the dark. Known quantities of ground leaf samples were extracted non-sequentially using 100% petroleum ether, acetone, methanol, ethanol and water. Different fractions were filtered using a Büchner filter and evaporated to dryness in a vacuum concentrator at 30 °C. The extracts were poured into pill vials, left to dry under a fan at room temperature and stored in a sterile container in a walk-in fridge before use. Antifungal activity was determined using the microdilution method as described by Clinical Laboratory Standards (2002) (Reference method for broth dilution antifungal susceptibility testing of filamentous fungi. Approved standard M38-A. National Committee for Clinical Laboratory Standards) with modifications. The dried extracts (25 mg each) were weighed into separate Eppendorfs and dissolved in 1 mL of 70% ethanol, sonicated and then used for antifungal bioassays. *Trichophyton rubrum* (ATCC 28188), *T. mentagrophytes* (ATCC 9533) and *Microsporum gypseum* (ATCC 24102) were purchased from Quantum Biotechnologies, Randburg, South Africa. They were grown on yeast malt agar (Y3127). A small portion of a seven-day-old culture was scraped with a scalpel into McCartney bottles containing yeast malt broth (Sigma Y3752) and incubated at 37 °C for 2 (*M. gypseum*) and 7 days (*T. rubrum* and *T. mentagrophytes*), respectively. The suspension was adjusted using a spectrophotometer to an absorbance of between 0.25 and 0.28 at 530 nm. The fungal inoculum was prepared with 1 × 10^6^ Colony Forming Units (CFU) in the suspension. Susceptibility testing was carried out using the micro-dilution method. Distilled water (100 μL) was pipetted into each of the 96 wells in a microtitre plate and 100 μL of the diluted leaf extract were added to the 1st well of the column and then serially diluted down the wells. The diluted fungal suspension (100 μL) was added to each of the 96 wells. The microtitre plates were subsequently incubated at 37 °C for 48 h after which 50 μL of *p*-iodonitrotetrazolium violet (INT) (Sigma 58030-1G-F) indicator was added to each well to determine growth inhibition. The microtitre wells were observed 30 min to 1 h after the indicator was added. The lowest concentration of clear wells was regarded as the minimum inhibitory concentration (MIC) values. Broth (50 μL) was added to each well immediately after the readings were taken. The microtitre wells were incubated at 37 °C for 48 h and then observed in order to determine the minimum fungicidal concentration (MFC). The assay was repeated three times in order to ensure consistency of results.

### Analysis and antifungal assay of essential oil (EO)

Leaves of *Coleonema album* and *C. pulchellum* were sourced (in September 2015) from the South African National Biodiversity Institute, Kirstenbosch. The leaves were freeze-dried and stored in sealed plastic bags. Essential oil was extracted from 20 g of freeze-dried leaves using hydrodistillation method. The extraction of oil was done according to the Czech Pharmacopeia. The EO derived was subjected to analysis using Gas Chromatography Mass Spectrometry (GCMS) (Agilent 5975C, Palo Alto, CA). Sample injection volume was 1 μL at 50 °C for 15 min, increased to 250 °C at 2 °C/min, held at 250 °C for 15 min (injection at 220 °C; column HP-5, 30 μm × 250 mm × 0.25 μm; carrier gas He 1 mL/min; MS detection). The major constituents (≥1.5%) were recorded individually while the minor constituents (≤1.5%) were recorded as ‘others’.

The antifungal activity of the EO derived from the leaves of the plants was analyzed using the volatile release plate method. This method was adopted to determine if gradual exposure to EO volatiles can effectively inhibit the growth of the fungi mycelia. Essential oil naturally is composed of several volatile components which evaporate under different temperatures. A variety of these components have been reported to inhibit the growth of infectious pathogens *in vitro*. *Trichophyton rubrum* (ATCC 28188) and *T. mentagrophytes* (ATCC 9533) were grown in yeast malt broth for one week and the fungal suspensions were subcultured on fresh yeast malt agar for one week. Mycelia of 0.3 cm diameter were taken from plates with good mycelia growth. The agar supporting the mycelia was sliced off and the mycelia were placed on fresh agar plates. Filter paper was placed on the upper lid of Petri dishes with fresh agar. The filter paper was impregnated with 20 μL of EO in such a way that the mycelia placed on the agar plate were directly exposed to the EO volatiles. Plates with similar diameter (0.3 cm) of *T. rubrum* and *T. mentagrophytes* without EO were used as controls. The plates were incubated for a week and the diameter of the mycelia was recorded after seven days of incubation. A formula, Fungal Growth Index (FGI) was used to calculate the increase in the size of the mycelia in order to be able to determine the extent of the inhibition of the fungi by the EO volatiles.

$$\text{FGI}=\frac{\left(\text{Final diameter of mycelia}-\text{Initial diameter of mycelia}\right)}{\text{Initial diameter of mycelia}}\times 100$$

The mycelia from each Petri dish were subcultured on fresh agar plates and incubated for another seven days at 37 °C in order to determine if the treatments had a fungistatic or fungicidal effect.

### Determination of oxygen radical absorbance capacity (ORAC)

Fresh plant material (leaves) was sourced from the South African National Biodiversity Institute, Kirstenbosch. The sample was freeze-dried, ground into powder and stored in air-tight containers at 23 ± 1 °C. The sample (100 mg) was dissolved in 1 mL of 80% methanol and sonicated for 30 min. It was subsequently filtered using a syringe filter. The antioxidant activity of the leaf extracts was determined using the ORAC method according to Ou et al. (2001). Briefly, 100 μL of 500 nM fluorescein and 25 μL of diluted extracts were pipetted into each working well of a microplate preincubated at 37 °C. Then, 25 μL of 250 mM 2,2′-azobis(2-amidinopropane) dihydrochloride (AAPH) was added and the microplate was shaken for 5 s on a shaker. The fluorescence (Ex. 485 nm, Em. 510 nm) was read every 3 min for 90 min. Net area under the curve was used to calculate antioxidant capacity which was expressed as Trolox equivalents.

## Results

### Antifungal assay of leaf extracts

The methanol extract of *C. pulchellum* (195 μg/mL), and methanol and acetone extracts of *C. album* (781 μg/mL) exhibited the best activity against *T. rubrum*, while the methanol extracts of *C. album* and *C. pulchellum* (391 μg/mL) exhibited the best activity against *T. mentagrophytes* (Table 1). *Coleonema pulchellum* and *C. album* methanol extracts (49 and 195 μg/mL, respectively) showed the best antifungal activity against *M. gypseum* (Table 1). Extracts with good antifungal activities against *M. gypseum* include: *C. album* acetone and ethanol extracts (391 μg/mL), *C. pulchellum* acetone and ethanol extracts (781 μg/mL). The methanol extract of *C. pulchellum* inhibited the growth of all the tested fungal strains with MIC values of 195, 391 and 49 μg/mL for *T. rubrum*, *T. mentagrophytes* and *M. gypseum*, respectively. The levels of antifungal activities recorded above are noteworthy as the values are less than 1000 μg/mL, similar to the MIC values of the antibiotics ketoconazole and griseofulvin used in this study. The water extracts of the leaves gave very high MIC values (results not shown). None of the extracts tested had a fungicidal effect.

**Table 1.** Antifungal activity of *Coleonema album* and *C. pulchellum* leaf extracts against *Trichophyton rubrum*, *T. mentagrophytes* and *Microsporum gypseum*.

		Petroleum ether (μg/mL)		Acetone (μg/mL)		Methanol (μg/mL)		Ethanol (μg/mL)	
Fungal strain	Plant species/test compound	MIC	MFC	MIC	MFC	MIC	MFC	MIC	MFC
*Trichophyton rubrum*	*Coleonema album*	3125	6250	**781**	6250	**781**	3125	1563	3125
	*Coleonema pulchellum*	3125	6250	3125	3125	**195**	3125	1563	6250
	Ketoconazole	>1000							
	Griseofulvin	>1000							
*Trichophyton mentagrophytes*	*Coleonema album*	3125	6250	1172	3250	**391**	3125	1563	6250
	*Coleonema pulchellum*	6250	6250	3125	6250	**391**	6250	1563	3125
	Ketoconazole	>1000							
	Griseofulvin	>1000							
*Microsporum gypseum*	*Coleonema album*	6250	6250	**391**	3125	**195**	3125	**391**	3125
	*Coleonema pulchellum*	6250	6250	**781**	3125	**49**	1563	**781**	3125
	Ketoconazole	<1000							
	Griseofulvin	>1000							

Bold values indicate the best antifungal activity.

### Analysis and antifungal assay of essential oil (EO)

Caryophyllene (24.91%), *trans*-β-ocimene (9.49%), β-myrcene (7.96%) and octahydro-7-methyl-3-methylene-4-(1-methylethyl)-β-copaene (7.53%) are the major components of *C. album* EO while bicyclo[3.1.0]hex-2-ene,4-methyl-1-(1-methyethyl)-β-phellandrene (32.04%), (+)-3-carene (10.88%), β-ocimene (9.46%), γ-elemene (8.95%) and α-pinene (7.43%) are the major components of *C. pulchellum* EO. The components of the oil greater than 1.5%, found in both EO of *C. album* and *C. pulchellum* are caryophyllene, bicyclo[3.1.0]hex-2-ene,4-methyl-1-(1-methyethyl)-β-phellandrene, α-pinene, γ-elemene and carene (Table 2). However, the common bioactive compounds exist in both EO in different quantities.

**Table 2.** *Coleonema album* and *C. pulchellum* essential oil bioactive compounds.

Bioactive compounds (%)	*Coleonema album*	*Coleonema pulchelum*
Α-Terpineol	3.69	ND
Bicyclogermacrene	5.11	ND
Bicyclo[3.1.0]hex-2-ene,4-methyl-1-(1-methyethyl)- β-Phellandrene	5.97	32.04
(+)-3-Carene	5.98	10.88
(+)-4-Carene	ND	3.76
Caryophyllene	24.91	6.98
Eucalyptol	2.58	ND
γ-Elemene	1.84	8.95
β-Myrcene	7.96	4.47
Octahydro-7-methyl-3-methylene-4-(1-methylethyl)- β-copaene	7.53	ND
β-Ocimene	ND	9.46
1,3,6-Octatriene,3,7-dimethyl-, (Z)-β-ocimene	2.25	ND
α-Phellandrene	ND	1.60
α-Pinene	ND	7.43
β-Phellandrene	ND	3.73
β-Pinene	7.00	5.36
*trans*-β-Ocimene	9.49	ND
Others	15.69	5.34

ND: not detected.

The EO volatiles from both plants inhibited the growth of *T. rubrum in vitro* with final mycelia diameter of 0.3 cm and FGI of 0% (Table 3) each. *Coleonema album* EO volatile had total inhibition on both *T. rubrum* and *T. mentagrophytes*. However, *C. pulchellum* EO volatile did not have total inhibition on the growth of *T. mentagrophytes* mycelia as the final mycelia diameter of *T. mentagrophytes* treated with the EO was 0.5 cm resulting into a 66.67% FGI. Overall, volatiles from *C. album* and *C. pulchellum* EO inhibited the growth of the fungi *T. rubrum* and *T. mentagrophytes* compared to the final diameter of the mycelia controls of 2.0 and 1.0 cm diameters, respectively.

**Table 3.** Effect of *Coleonema album* and *C. pulchellum* EO volatiles on Fungal Growth Index (FGI) (%) of *Trichophyton rubrum* and *T. mentagrophytes* as compared to the control.

Fungal strains	*Coleonema album* oil FGI (%)	*Coleonema pulchellum* oil FGI (%)	Control FGI (%)
*Trichophyton rubrum* (diameter) cm	0	0	566.67
*Trichophyton mentagrophytes* (diameter) cm	0	66.7	233.33

### Determination of ORAC

The antioxidant activity expressed as Trolox equivalents (μmol Trolox Equivalents/g) of *C. album* and *C. pulchellum* leaf extracts are recorded in Table 4. The leaf extracts of *C. pulchellum* has higher Trolox equivalents value (1126.7 ± 68.1 μmol Trolox Equivalents/g) compared to that of its sister species *C. album* (942.2 ± 34.9 μmol Trolox Equivalents/g).

**Table 4.** ORAC^FL^ values of *Coleonema album* and *C. pulchellum* expressed as Trolox equivalents.

Plant species	μmol Trolox Equivalents/g
*Coleonema album*	942.2 ± 34.9
*Coleonema pulchellum*	1126.7 ± 68.1

## Discussion

Several species of the Rutaceae family have been used in traditional medicine and coumarins could be responsible for a majority of their activities (Gray & Waterman 1978). Extracts of *C. album* displayed potent and relevant pharmacological activities, with a considerable antifungal activity against several strains responsible for important infectious diseases (Esterhuizen et al. 2006a). The compounds from *C. album* could be of great pharmaceutical interest for therapeutic application as complementary antifungal agents (Esterhuizen et al. 2006a). Its sister species, *C. pulchellum* contains phenylpropenes, phenylpropanoids and terpenoids which exhibit antimicrobial properties (Brader et al. 1997).

In this study, the methanol extract of *C. pulchellum* demonstrated good *in vitro* activity against the three fungi tested indicating the potency of the plant against skin diseases of fungal origin. Hence, *C. pulchellum* (methanol extract) has good prospects for the formulation of an effective cure for skin disease. Brader et al. (1997) attributed the observed antimicrobial activity to the reactivity of the phenolic hydroxyl group. The plant *C. pulchellum* contains biologically potent therapeutic phytochemicals with high antibacterial and antioxidant activities (Baskaran et al. 2014). The result of this study support the report of Baskaran et al. (2014) as the leaf extracts of *C. pulchellum* displayed a remarkable antioxidant activity much higher than that of *C. album* leaf extract. *n*-Hexadecanoic acid, a compound isolated from *C. album*, has been identified as one of the compounds that possess fungistatic properties against mammalian skin pathogens (Esterhuizen et al. 2006a). It has been reported to possess antifungal activity against *Trichophyton mentagrophytes*, a zoophilic skin fungus which is responsible for athlete’s foot (Wood & Weldon 2002). In this study, methanol extract and EO of *C. album* exhibited good activity against *T. mentagrophytes* and thus confirm the reports of the cited authors.

Essential oils are known to have several properties which are of great importance in skin health (Rhind 2012). Such properties include skin barrier function, maintenance and restoration of texture and hydration levels, reduction of inflammation, stimulation of cell regeneration, prevention or control of infections and allergic responses, and alleviation of itching (Rhind 2012). Antimicrobial activities of EO have been attributed mainly to its volatile components such as monoterpenes which in many cases are the main components of EO. The EO extracted from the epicarp of *Citrus sinensis* exhibited absolute fungitoxicity against 10 post-harvest pathogens (Sharma & Tripathi 2006). *Haplophyllum tuberculatum* oil affected the mycelial growth of *Curvularia lunata* and *Fusarium oxysporum* (Al Burtamani et al. 2005). In this study, the volatiles of *C. album* and *C. pulchellum* EO displayed antifungal activity against two important skin fungi, *T. rubrum* and *T. mentagrophytes.* It is, however, not certain which component(s) of the EOs are responsible for the activity.

The major components of *C. album* oil are β-phellandrene (29.1%) and myrcene (20.5%) among the 43 components characterized (Başer et al. 2006). However, the major components of the *C. album* oil analyzed in this study are caryophyllene (24.91%), *trans*-β-ocimene (9.49%), β-myrcene (7.96%) and octahydro-7-methyl-3-methylene-4-(1-methylethyl)-β-copaene (7.53%). Bicyclo[3.1.0]hex-2-ene,4-methyl-1-(1-methyethyl)-β-phellandrene (32.04%), (+)-3-carene (10.88%), β-ocimene (9.46%), γ-elemene (8.95%) and α-pinene (7.43%) are the major components of *C. pulchellum* EO analyzed in this study.

The literature available on the analysis of *C. pulchellum* EO, however, differ from what we found in this study as bicyclo[3.1.0]hex-2-ene,4-methyl-1-(1-methyethyl)-β-phellandrene (32.04%) form one-third of the bioactive compound composition. Essential oil from the leaves of Australian *C. pulchellum* was found to consist mainly (50%) of monoterpene hydrocarbons (Brophy & Lassak 1986). Monoterpene composition of the oil of South African *C. pulchellum* is more than that of the Australian sample (Brophy & Lassak 1986). In the three oil samples of *C. pulchellum* analyzed by Başer et al. (2006), monoterpenes dominated with 92.2%, 81.4% and 97.0%, respectively. The presence of β-phellandrene and sabinene as part of the major compounds was the main difference in two of the samples while β-pinene, linalool and α-pinene form part of the major components in all the three samples analyzed. Overall, monoterpenes were the major compounds of all the oils ranging from 86.5% to 97.3% (Başer et al. 2006).

Harvesting related factors such as season of harvest, geographical location and age of the plant at the time of harvest plays crucial roles on the EO yield and most importantly the composition of the bioactive compounds present in the oils (Preedy 2015). Cavacrol, the major component of oregano EO was higher during the drier and warmer season (70.75–84.88%) while several other compounds accumulated at higher levels during the wetter and colder season along with very low levels of cavacrol (56.46–75.12%) (Karamanos & Sotiropoulou 2013). The season of harvest, plant age and geographical location could be some of the factors responsible for the chemical composition of *C. album* and *C. pulchellum* EO in this study and the antioxidant activity of their leaf extracts.

There is increasing interest in the use and quantification of antioxidant capacity in the pharmaceutical and cosmetic industries (Ou et al. 2001). This interest results from the enormous evidence of the importance of reactive oxygen/nitrogen species (ROS/RNS) in aging and pathogenesis of diseases (Halliwell & Aruoma 1991; Wink et al. 1991; Nguyen et al. 1992; Stadtman 1994). Natural antioxidants, such as EOs seem to have the potential to offer considerable protection against oxidative skin damage (Rhind 2012). Caryophyllene and limonene have been reported to display strong 5-lipoxygenase inhibitory activity (Baylac & Racine 2003).

Several EOs have antioxidant and anti-inflammatory activities, and some appear to be significant, specifically in relation to the skin as there is a possibility that antioxidant activity is linked with keratinocyte differentiation, and hence barrier function and texture (Rhind 2012). This implies the crucial role oxidative stress plays in the development of AD. Antioxidant activity is one of the major and most important biological activities attributed to EOs and is at the root of many other properties which include anti-inflammatory activity (Rhind 2012). Essential oils have been reported to have anti-inflammatory activities and their constituents such as terpene hydrocarbons, sesquiterpene hydrocarbons and sesquiterpene alcohols have been reported to display 5-lipoxygenase inhibitory activity (Alexander 2001; Baylac & Racine 2003). The hydrophobic nature of EO makes it difficult to determine its antioxidant activity of EO *in vitro*. Leaf extracts of *C. album* and *C. pulchellum* were used instead of their EOs.

In this study, *C. pulchellum* leaf extracts gave higher antioxidant activity compared to *C. album* extract. The antioxidant activity in an array of different natural sources is often attributed to coumarins (Moure et al. 2001; Heim et al. 2002). *Coleonema album* extracts possessed significant *in vitro* antioxidant activity which could be as a result of the phenolic compounds present in the plant extract (Esterhuizen et al. 2006b). Coumarins and flavonoids are phenolic compounds and have been demonstrated to have potent antioxidant activities as a result of their phenolic hydroxyl groups (Demmig-Adams & Adams 2002) and an ideal structural chemistry for free radical scavenging activity (Rice-Evans & Burdon 1994). The phenolics have received increased attention as a result of the various scientific reports indicating a correlation between consumption of food and beverages rich in phenolics and reduced incidence of diseases linked to oxidative stress (Rice-Evans & Burdon 1994; Martinez-Cayuela 1995; Heinecke 1998; Chisolm & Steinberg 2000).

Under various pathological conditions, an oxidative cascade may be generated which can induce cytotoxicity and apoptosis and may have a significant role in inflammation, enhancing the release of cytokines and modifying lipoproteins to pro-inflammatory forms (Gutteridge 1988; Deigner & Hermetter 2008). Lipid peroxidation (LPO) products react with sugars, proteins, and DNA (Niki 2009). The end products of LPO, such as malondialdehyde, 4-hydroxy-2-nonenal (4-HNE) and 4-hydroxy-2-hexanal (4-HEE) can damage proteins by reacting with various amino acids both *in vivo* and *in vitro* (Catalá 2009). Oxidative stress causing the production of lipid peroxides may thus be a major factor in the development of skin inflammatory diseases (Braconi et al. 2010; Sivaranjani et al. 2013).

Generally, it is recognized that a combination of environmental factors such as allergens, microbial infection and genetic factors induce the multiple immunologic and inflammatory responses seen in AD patients (Lü et al. 2009). Atopic dermatitis (eczema/ringworm) can affect any part of the human body but seems to occur more frequently on the hands and feet, ankles, face, wrists, upper chest and neck (Sivaranjani et al. 2013) and the skin around the eyes and the eyelids (Thestrup 2000). Eczema is a form of chronic inflammatory skin disease (Wagener et al. 2013) with various hallmarks, such as chronic relapsing of skin inflammation, disturbance of the epidermal-barrier function which at its climax leads to dry skin and eruptions (Sivaranjani et al. 2013). In an attempt to achieve an increased therapeutic rate of treatments of skin diseases, a combination of topical and oral anti-inflammatory drugs have been used (Soares et al. 2013). A decrease in inflammation and an improved rate of recovery could be achieved in the treatment of inflammatory skin conditions by targeting oxidative stress (Wagener et al. 2013). Atopic dermatitis is associated with an impaired oxidative status, and systemic alterations in antioxidant patterns of the skin have been found in skin of AD patients as well as in non-lesioned skin as an adaptive response to chronic inflammation of the epidermis (Briganti & Picardo 2003). Filament aggregating protein (filaggrin, FLG) is a key protein that plays a major role in the formation of the cornified cell envelope, which is crucial for an effective skin barrier (Candi et al. 2005). Studies have shown that loss-of-function (null) mutations in the gene-encoding FLG leads to skin barrier impairment in AD patients (Marenholz et al. 2006; Palmer et al. 2006; Weidinger et al. 2006). Many AD patients acquire a deficiency of filaggrin with subsequent barrier disruption as a result of the local inflammatory immune response (Howell et al. 2009).

A combination of events such as transepidermal water loss, disruption of the skin epidermal barrier system, oxidative stress, inflammation and ultimately, invasion by microbial pathogens are responsible for the manifestation of skin diseases. In view of this, an effective antifungal should encompass all the activities needed to restore skin health. As a result of the principal role of ROS in inflammatory pathologies, the restoration of redox balance forms a ground-breaking therapeutic target in the development of new approaches for the treatment of inflammatory skin conditions (Wagener et al. 2013). Nuclear factor E2-related factor 2 (Nrf2) links antioxidant defence mechanisms in the epidermis with the control of skin permeability barrier and antimicrobial defence (Schafer et al. 2012). The production of ROS and ROS-induced oxidation results in the disruption of the skin barrier (Thiele 2001). This implicates that restoration of redox balance through the use of effective antioxidant formulations in combination with an effective antifungal could ameliorate skin inflammation, reduce transepidermal water loss and thus restore the skin barrier function and prevent further invasion by microbial pathogens.

## Conclusions

Methanol extracts of *C. album* and *C. pulchellum* leaves contain bioactive compounds capable of inhibiting the growth of *T. rubrum*, *T. mentagrophytes* and *M. gypseum*. Hence, the extracts can provide a novel source of antifungal for the treatment of skin diseases. The EO of both species is rich in terpenes which have been reported in literature to exhibit antifungal activity. However, the season of harvest of the leaves will determine the level of antifungal activity displayed by the EO as bioactive compounds present in EO varies with environmental and physiological (age) factors during harvest of leaves or plant part(s). The EO of both plant species can be combined to formulate an effective antifungal ointment that can combat skin diseases associated with *T. rubrum*. The result of this study revealed the therapeutic value of *C. pulchellum*, which is not as popular as its sister species, *C. album*. Extracts and EO of *C. pulchellum* should be considered in the production of skin and hair care products just as its sister species (*C. album*). A combination of the antifungal and antioxidant activities of *C. album* and *C. pulchellum* leaf extracts and the antifungal activity of their EO volatiles against *T. rubrum* and *T. mentagrophytes in vitro* as reported in this study gives hope for the development of an effective and cheap skin antifungal of natural origin. The results of this study give credence to the incorporation of *C. album* extracts and EO into skincare products. As a result of the remarkable antifungal and antioxidant activities of *C. pulchellum*, as revealed in this study, the plant can be incorporated into skincare products just like *C. album*. However, toxicity studies of *C. pulchellum* extracts and EO need to be carried out in order to ensure it is safe for topical application on the skin.
